# Hydatid Cyst of the Inguinal Region: An Exceptional Localization

**DOI:** 10.7759/cureus.53471

**Published:** 2024-02-02

**Authors:** Mokhtari Omar, El mahjoubi Sohaib, Adnane Lachkar, Najib Abdeljaouad, Hicham Yacoubi

**Affiliations:** 1 Traumatology and Orthopedics, Centre Hospitalier Universitaire Mohammed VI, Oujda, MAR; 2 Traumatology and Orthopedics, Faculty of Medicine and Pharmacy, Mohammed First University, Oujda, MAR

**Keywords:** pelvic mri, pectineus muscle, pericystectomy, echinococcus granulosus larvae, inguinal region, cyst hydatid

## Abstract

Cystic echinococcosis (CE), stemming from the larval stage of the cestode *Echinococcus granulosus*, stands as a widespread parasitic zoonosis primarily afflicting the liver and lungs. However, instances in the inguinal region are exceptionally infrequent. We present a distinctive case involving a 49-year-old female with a progressively enlarging inguinal mass over a five-year period, characterized by the absence of hepatic or pulmonary involvement. This case underscores the unique clinical presentation and diagnostic intricacies associated with extrahepatic and extrapulmonary expressions of CE.

The presented case contributes to advancing our comprehension of unconventional hydatid disease presentations, highlighting the imperative for a multidisciplinary approach in both diagnosis and treatment. Ongoing research endeavors and collaborative efforts are pivotal for refining strategies and enhancing outcomes in patients with rare manifestations such as inguinal hydatid cysts.

## Introduction

Cystic echinococcosis (CE), resulting from the larval stage of the cestode *Echinococcus granulosus*, stands as a widespread parasitic zoonosis [1]. Also known as hydatid disease, this condition poses a significant health concern, particularly in developing nations, with a predilection for affecting the liver and lungs [2].

While the manifestation of hydatid cysts in the inguinal region is exceedingly uncommon, we present a rare case of an inguinal hydatid cyst with no concurrent hepatic or pulmonary involvement. This case underscores the unique clinical presentation and diagnostic challenges associated with extrahepatic and extrapulmonary occurrences of CE.

## Case presentation

A 49-year-old female, residing in a rural setting with an unremarkable medical history, sought evaluation at our university hospital for a progressively enlarging mass in her left inguinal region that had been developing over the past five years, with no associated history of trauma.

**Figure 1:**
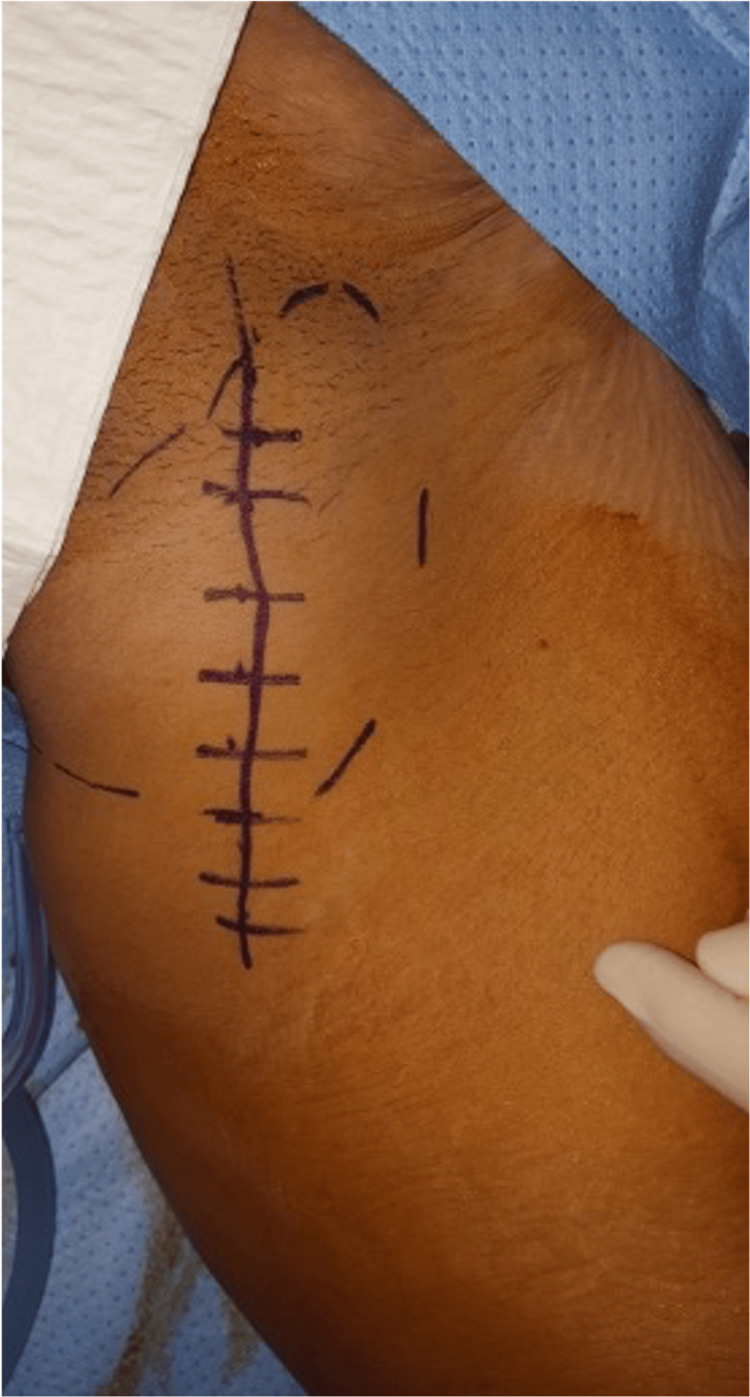
Clinical illustration of a 12-cm inguinal mass in its maximum dimension

Throughout this duration, the patient reported no fever or weight loss. Clinical examination revealed a rounded, soft mass measuring 12 cm in its largest dimension within the inguinal region (Figure 1).

The mass, non-painful upon palpation, exhibited mobility in both superficial and deep planes, devoid of signs of inflammation. Left hip mobility remained unrestricted, and neurological assessment yielded normal results. A general physical examination, liver profile, and laboratory tests revealed no abnormalities.

Chest X-rays and abdominal ultrasonography returned normal findings, while pelvic X-rays showed no anomalies.

**Figure 2:**
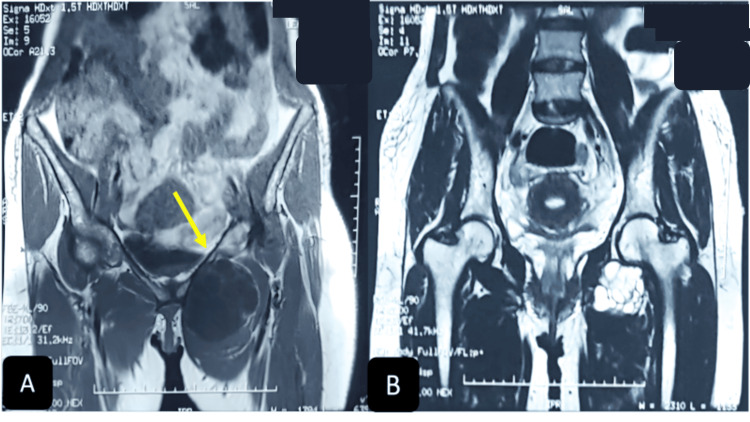
Coronal MRI section depicting a left inguinal mass involving the pectineus muscle, exhibiting hypo T1 signal (A) and hyper T2 signal (B), containing thin-walled vesicles that do not enhance after injection.

**Figure 3:**
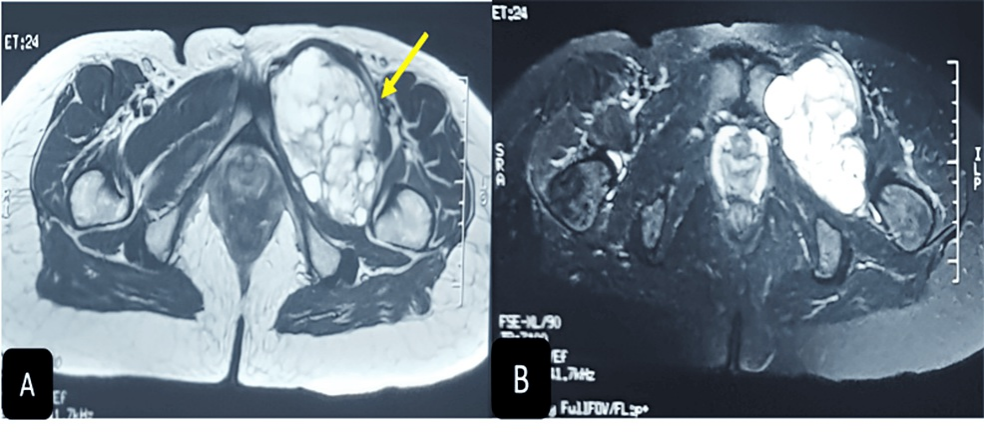
Axial section of the MRI; T1 (A) and T2 (B) signals are visualized

Magnetic resonance imaging (MRI) depicted a left inguinal mass exhibiting a hypo-T1 hyper-T2 liquid signal persisting on the fat saturation (FAT-SAT), measuring 101 x 68 mm and extending approximately 89 mm. The mass was delineated as multi-vesicular, thin-walled, with peripheral contrast. Notably, the mass exhibited a spatial relationship respecting the hip joint, relying on the muscular (pectineus muscle), compressing the left femoral pedicle, and occupying the subcutaneous compartments without extending into the abdominal region (Figures 2-3).

**Figure 4:**
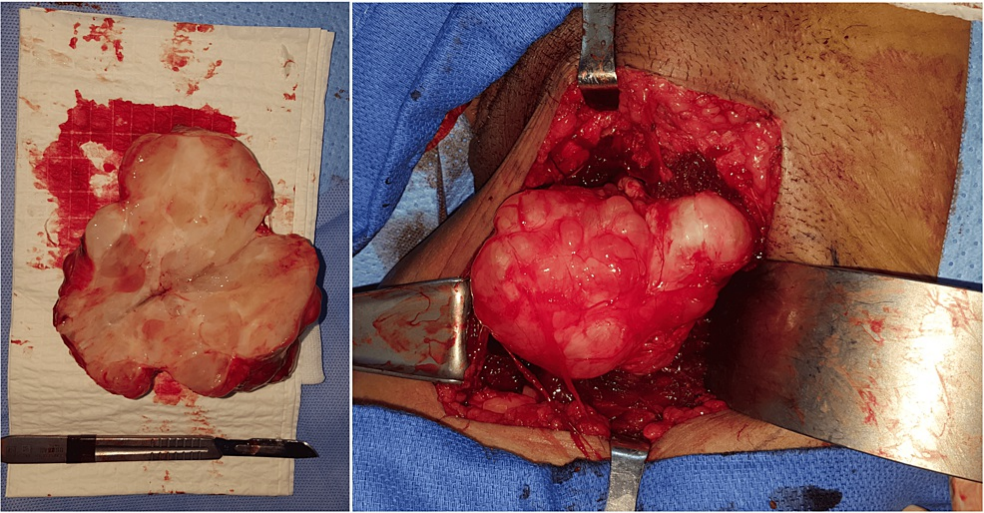
Intraoperative images illustrating the macroscopic appearance of the hydatid cyst

The thoracoabdominal computed tomography (CT) scan revealed no engagement of adjacent areas, despite a positive result in the hydatid serological test. Employing an anterior surgical approach, the mass was investigated, revealing a cyst situated in proximity to the muscles of the medial compartment of the left thigh (pectineus muscle; the adductor longus). The cyst presented as a multi-lobulated mass in the medial inguinofemoral region. A comprehensive pericystectomy was executed without jeopardizing the cyst's integrity (Figure 4).

To safeguard the surgical field edges, compresses soaked in a hypertonic saline solution were utilized. After cyst removal, surgical specimens underwent anatomopathological examination, confirming the diagnosis of a hydatid cyst. The patient's recovery post-surgery was uneventful, and albendazole treatment was started on the third day, continuing for six weeks to prevent recurrence.

During a five-year follow-up, there was no recurrence of the cyst locally or distantly, and the serological status remained negative.

## Discussion

Echinococcosis represents a parasitic infection affecting both humans and specific mammals. Dogs serve as the definitive host, while sheep function as the intermediate host. Human beings become infected by ingesting either cysts present in undercooked meat or oocysts present on vegetables, fruits, or hands soiled with soil. Contamination through drinking water is also possible [3].

Hydatid disease, attributed to *Echinococcus granulosus*, is an exceptionally rare occurrence in the inguinal canal. This disease spans global prevalence and currently impacts approximately one million individuals worldwide. Certain regions in South America, Africa, and Asia experience rates of up to 10% within specific populations. Hydatid disease is endemic in regions associated with cattle and sheep breeding, including central Europe, Mediterranean countries, the Middle East, South America, Australia, New Zealand, and South Africa [4].

The lifecycle involves the ingestion of parasite larvae, which penetrate the intestinal wall, enter circulation, and, via the portal vein, reach the liver. Within the hepatic sinusoids, most larvae are intercepted. However, a fraction may traverse the liver (the first filter) and reach the lungs (the second filter) and systemic circulation, giving rise to hydatid disease in diverse organs and sites. Dissemination through lymphatic channels has also been proposed as a plausible mechanism, accounting for cases featuring solitary cysts in unusual sites [5].

Conventional radiography often yields unremarkable results. The predominant diagnostic modality continues to rely on ultrasound, which exhibits heightened sensitivity in typical cases. However, unconventional presentations may manifest, wherein the lesion may demonstrate aberrant characteristics with or without internal echogenicity [6].

Magnetic resonance imaging is esteemed as the most dependable technique for assessing sizable cysts. It adeptly evaluates the cyst's dimensions, interfaces with adjacent tissues, and impacts neurovascular structures [7].

The optimal therapeutic approach involves complete surgical excision of the intact cyst, thereby averting the risk of cyst content leakage. In instances where complete excision is not feasible, intraoperative removal of the cyst contents and irrigation of the cyst pouch with scolicidal solutions are viable alternatives [8, 9].

Until the 1980s, the puncture of hydatid cysts was generally discouraged due to the associated risks of leakage and anaphylaxis. Akhan et al., recognized as pioneers in percutaneous treatment methods for hydatid cysts, have emphasized that percutaneous therapy represents a highly reliable and effective treatment modality. They advocate considering percutaneous therapy as a compelling alternative to surgical interventions [10].

Regardless of whether patients with hydatid cysts undergo surgical or percutaneous treatment, they necessitate a medical therapy regimen comprising scolicidal drugs and prophylactic antibiotics, administered both pre- and post-procedure. Mebendazole and albendazole, both belonging to the benzimidazole derivative class, are the most commonly employed scolicidal drugs. These medications function by impeding the parasite's glucose utilization, thereby hindering the formation of adenosine triphosphate [11].

## Conclusions

In conclusion, our case presentation of an inguinal hydatid cyst, free from hepatic or pulmonary involvement, underscores the exceptional rarity of hydatid cysts in the inguinal region. The successful management of this uncommon presentation employed a meticulous anterior surgical approach, leading to a complete pericystectomy without compromising the cyst's integrity.

Furthermore, the exploration of alternative treatment approaches, such as the puncture-aspiration-injection method with sclerosing agents, highlights the dynamic evolution in therapeutic strategies for muscular or subcutaneous hydatid cysts.
